# Rethinking Access in BEVAR: Single-Center Experience of the Feasibility of Upward-Facing Branches

**DOI:** 10.3390/jcm14176106

**Published:** 2025-08-29

**Authors:** Philipp Franke, Imam Tongku Padesma Ritonga, Bachar Al Haj, Yousef Shehada, Martin Austermann, Marco Virgilio Usai

**Affiliations:** Department of Vascular and Endovascular Surgery, St. Franziskus-Hospital Münster, 48145 Münster, Germany

**Keywords:** upward-facing branch, retrograde branch, BEVAR, FEVAR, aortic aneurysm

## Abstract

This is a single-center study about upward facing in branched endovascular aortic repair. **Background**: The evolution of branched endovascular aortic repair (BEVAR) has introduced upward-facing branches as a novel approach to facilitate exclusive transfemoral access in complex aortic aneurysm repair. This study evaluates the feasibility, safety, and early outcomes of custom-made BEVAR devices incorporating upward-facing branches in patients with cranially oriented renal arteries. The investigation further aims to analyze the technical success and mid-term outcomes related to these novel devices, as well as to identify any challenges or complications specific to the use of upward-facing branches in clinical practice. **Methods**: We retrospectively analyzed 17 patients treated at a single center between January 2020 and December 2024 using custom-made Cook Medical branched stent grafts with at least one upward-facing branch. Demographics, comorbidities, target vessel details, bridging stent graft (BSG) configurations, and procedure-related complications were collected. The primary endpoints were technical success and branch patency. Secondary endpoints included short- and mid-term branch-related complications. **Results**: The cohort had a mean age of 70 years, with hypertension (88%) and coronary artery disease (47%) being common comorbidities. Technical success was achieved in 100% of cases. The left renal artery was the most frequently targeted vessel (63.2%). Most upward-facing branches were bridged using a combination of balloon-expandable and self-expandable stents. One patient (5.9%) experienced a renal bleeding complication requiring embolization. There were no cases of primary stent occlusion or dislocation. At a mean follow-up of 14 months, one asymptomatic occlusion of an upward-facing branch was detected in computed tomography angiography. No further upward-facing branch-related complications occurred, and 1-year follow-up was available in 41.2% of patients. **Conclusions**: In our single-center study including 17 patients, upward-facing branches in BEVAR demonstrate high technical success and a low complication rate, offering a promising alternative to traditional access strategies. These findings support broader adoption in select anatomical scenarios, pending larger comparative studies and longer-term data collection.

## 1. Introduction

In the early stages, branched endovascular aortic repair (BEVAR) was a treatment option for older and multimorbid patients with thoracic abdominal aortic aneurysms, offering a less invasive approach than open repair, but is nowadays offered to different patient groups and has become the first-line option for the treatment of complex aortic aneurysms in many parts of the world. Over the years, advancements in device design and surgical technique have significantly improved the outcomes and expanded the indications for BEVAR, making it accessible for a wider patient population. Studies have shown that the target vessel orientation, especially the orientation of the renal arteries, plays a crucial role for technical success of the procedure [1,2,3].

The bridging to the target vessel plays an important role for the success of the treatment when it comes to BEVAR. The length and tortuosity of the branches have an influence on branch instability, and the decision regarding which bridging stents (BSGs) are used, balloon-expandable covered stent alone or a combination of balloon-expandable covered stents and self-expandable covered stents, is related to patency rates [4,5].

Furthermore, precise planning and imaging techniques have enhanced the ability to predict and mitigate potential complications related to branch placement and stent durability. In our standard BEVAR routine, we connect the downward-facing branches through a transaxillary approach to the target vessel. Upward-facing branches could simplify these complex procedures by being canulated from a transfemoral approach and may also help to connect cranially oriented target vessels [1]. This new approach potentially reduces procedural complexity and patient morbidity by eliminating the need for additional vascular access points and thereby minimizing the risk of access-related complications.

Chamseddin et al. showed in a multicenter study that a transfemoral-access-only approach was associated with fewer perioperative cerebrovascular events than in procedures with upper-extremity access. This study concludes that an upper-extremity access is considerable in complex treatments because of its overall acceptable risk for perioperative cerebrovascular events. But a transfemoral-access-only approach should be preferred when possible [6].

Upward-facing branches could be a solid way to solve some of the addressed problems. They can be easily reached from a transfemoral approach, and they could help to avoid the kinking of BSG with the risk of occlusion when a cranially oriented target vessel needs to be connected. But they must prove equal patency and technical success rates in comparison to downward-facing branches. Data about the use of cranially oriented branches in BEVAR is missing, as far as the authors know.

The aim of this single-center study was to evaluate the technical and clinical implications of using upward-facing branches, especially with regard to technical success and primary patency. For this study, we enrolled every consecutive patient who received a custom-made branched endovascular aortic repair between January 2020 and December 2024, independent of the underlying aortic pathology, with at least one upward-oriented target vessel.

## 2. Methods

This is a single-center study about patients with complex thoracoabdominal aortic aneurysms who were treated with a custom-made branched endovascular aortic repair (BEVAR) in our center, with at least one upward-facing branch, between January 2020 and December 2024. An upward-facing branch was planned for patients with cranially oriented target vessels (<90 degrees to the axis of the aorta).

Demographics, comorbidities, target vessels, the number and combination of bridging stent grafts (BSGs), as well as upward-facing branch-related complications, were collected and analyzed retrospectively. Data quality was ensured by rigorous chart review and follow-up documentation. The median follow-up was eight months. There was one loss of follow-up after discharge from the hospital.

The small number of patients and the absence of a comparison group hinders the use of statistical tests. We used percentages and means.

This study complies with the Declaration of Helsinki. Local regulatory approvals were waived due to the retrospective design of the study and the absence of patient identifiable details being shared between the collaborators. All patients undergoing aortic repair in our institution signed to be included in a prospective database, with the aim of performing retrospective studies through it.

### 2.1. Pre- and Perioperative Management

**Figure 1:**
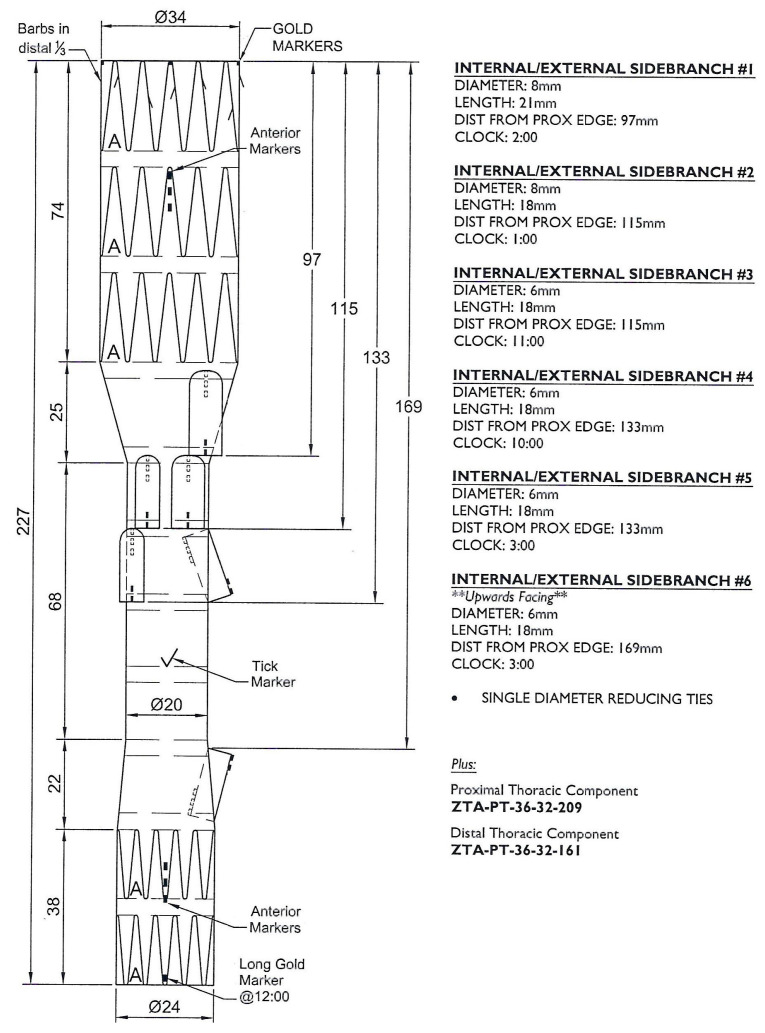
Planning of a custom-made device with one upward-facing branch.

Every patient received a preprocedural thin-slice computed tomography angiography (CTA), and the measurement and planning of the devices was performed by the operating vascular surgeon in cooperation with the custom-made device planning center from Cook (Bloomington, IN, USA) (Figure 1). This multidisciplinary approach ensures an optimal device fit and minimizes the risk of procedural complications.

Indication for an upward-facing branch was the upward orientation of the target vessel (<90 degrees, relative to the axis of the aorta) to facilitate cannulation.

After the operation or before discharge, every patient received a CTA to control the result. This postprocedural imaging is essential for early detection of complications and to verify the device positioning.

In most cases and the absence of contraindications, the patient received a combination of anticoagulation and antiplatelet therapy, according to local protocol, with aspirin 100 mg and clopidogrel 75 mg once a day after the operation for 3 months, followed by aspirin alone quoad vitam. A multicenter study of Nana et al. compares single anti-platelet therapy (SAPT) vs. dual anti-platelet therapy (DAPT) in F/BEVAR and concludes that DAPT is associated with better outcomes, referring to target vessel patency, among other things [7]. According to the patient’s comorbidities, variations of the local protocol are possible.

To reduce the risk of paraplegia, every patient was invasively monitored at our intensive care unit (ICU) for at least 48 h, keeping the mean arterial pressure above 80 mmHg and with a Hemoglobin value above 10 gr/dl. Meticulous ICU management is key in preventing neurological complications.

### 2.2. Technical Aspects

In every case, a custom-made device by Cook Medical (Bloomington, IN, USA) was used. The procedures were performed under general anesthesia.

Advanced imaging and navigation techniques were employed to ensure precision. All procedures of this cohort were performed with the use of the Fusion technique. Our standard approach contains a bilateral percutaneous transfemoral access. For the preclosure of the common femoral artery, we use ProStar XL by Abbott (Abbott Park, IL, USA). The access to the left axillar artery is accomplished by open surgical exposure. When the main prothesis is implanted, we close the bilateral transfemoral access to have good inflow for both hypogastric arteries, and then start to connect the branches to the target vessels. We use a combination of a 12 French and an 8 French Sheath by Cook (Bloomington, IN, USA) to gain stability and always start to connect the superior mesenteric artery, afterwards the renal arteries, and then the celiac trunk. For canulation of the branches and the target vessels, we normally use a Berenstein catheter by Merit (South Jordan, UT, USA), and then use a Rosen guidewire by Cook (Bloomington, IN, USA) for the deployment of the BSGs. After the deployment of the ballon expendable covered stents, we dilatate them with eight atmospheres in a first step, and then in a second step, flair them with fourteen atmospheres around the inner branch ring. These precise technical steps are important for optimal device function and long-term success.

For the bridging of the upward-facing branches, we must adjust our procedure. In these cases, we canulate and bridge the upward-facing branches from the transfemoral access first and close the bilateral access afterwards. This modification enhances procedural efficiency and safety.

The target vessels were connected to the upward-facing branch with BSGs, combining balloon-expandable covered stents and/or self-expandable covered stents through the transfemoral accesses. The choice of the combination and number of BSGs depends, on the one hand, on the distance between the branch and the target vessel and on the landing zone in the target vessel, and on the other hand, on the intraoperative performed angiography. The downward-facing (“normal”) branches were connected to their target vessels from a left axillar access. The celiac trunk and the superior mesenteric artery were always connected through downward-facing branches.

**Figure 2:**
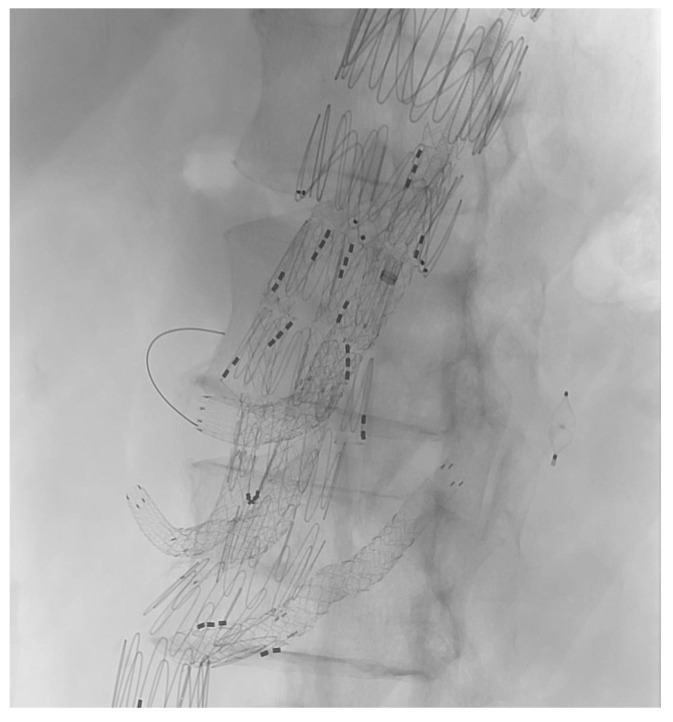
XA during the procedure.

The target vessel of the upward-facing branch was always at least one of the renal arteries (Figure 2). In two cases, both renal arteries were connected through upward-facing branches, and in one case, we used it for an accessory renal artery.

**Figure 3:**
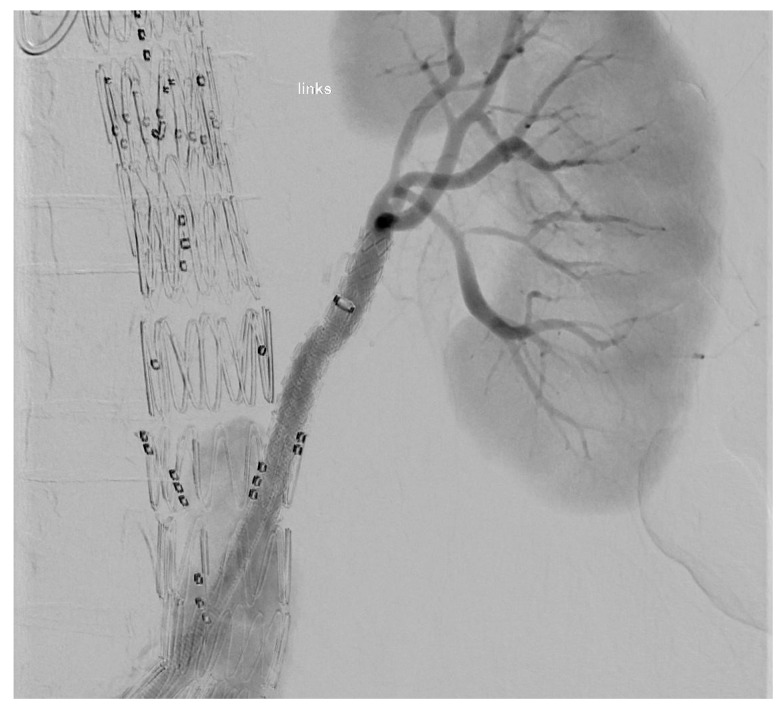
Upward-facing branch for the LRA.

**Figure 4:**
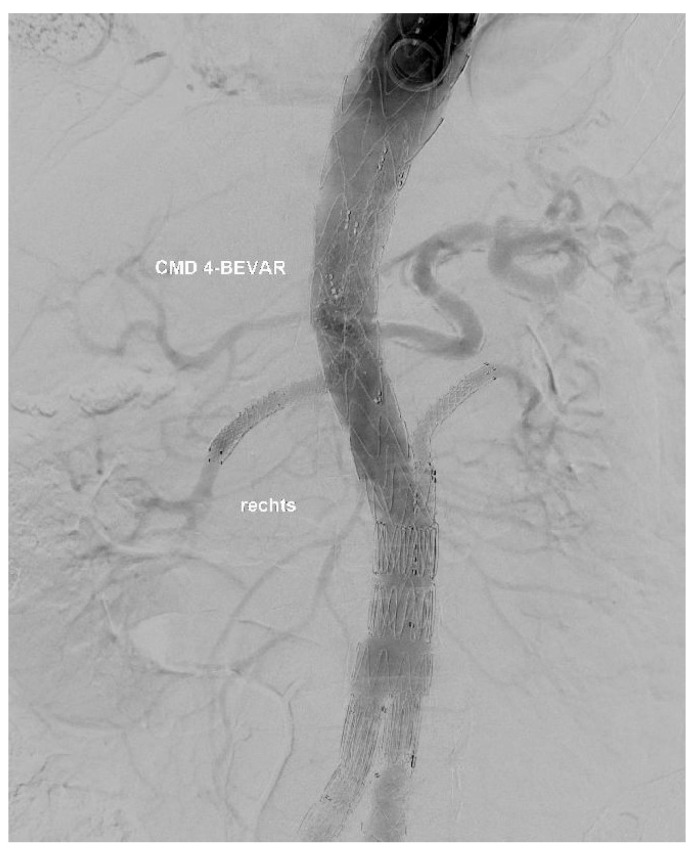
Result of a procedure.

At the end of every operation, an angiography was performed to obtain an overview regarding if the BSGs are patent and if there is any sign of an endoleak of any type (Figure 3 and Figure 4).

### 2.3. Endpoints

The primary endpoints were the technical success of the whole procedure (freedom from a type Ia endoleak, branch patency, good placement of the branched device) itself and relating to use of the upward-facing branches, defined as primary patency (intraoperative angiography and postoperative CTA) and the absence of any branch-related complications like a type III endoleak or stent migration during the procedure. Short-term (30 days to six months) and mid-term (above six months) target vessel instability (defined as occlusion, stenosis, endoleak type Ic/IIIc, and re-intervention) and patency were secondary endpoints.

## 3. Results

We enrolled 17 patients. Ten of them were male (59%) and seven female (41%), with a mean age of 70.29 ± 6.06 years. The most common comorbidities were hypertension in 15 patients (88%) and coronary artery disease in 8 patients (47%). In total, 47% (eight patients) of the cohort had dyslipidemia and 29% (five patients) COPD. Three patients (18%) had a smoking history, and one patient (6%) was an active smoker. Seven (41%) patients received a previous aortic repair. In particular, six of them (35%) were treated with a TEVAR before the index-intervention, and two (12%) of them had a frozen elephant trunk operation (one patient received FET and TEVAR). An overview of the demographics and comorbidities of the patients can be found in Table 1.

Thoracoabdominal aortic aneurysm (TAAA) type II (six patients, 35.3%) and type IV (six patients, 35.3%) were the most common causes for the intervention, while the mean diameter of the aneurysms was 60 ± 7.24. Three patients (17.8%) had a type III TAAA and one (5.8%) a type V TAAA. In one case, a penetrating aortic ulcer (PAU) was diagnosed with a diameter of 38 mm. We planned a BEVAR with four branches for 76.5% of the cohort (13). Four (23.5%) received a BEVAR with five branches. All cases were performed electively and treated with a custom-made device by Cook Medical. Table 2 breaks down the anatomic information and the number of target vessels.

Fifteen (88.2%) of the used devices were planned with one upward-facing branch and two (11.8%) with even two upward-facing branches. The commonest target vessel of the upward-facing branch was the left renal artery (12, 63.2%). In six cases (31.6%), the right renal artery was bridged through an upward-facing branch, and in one case (5.2%), we planned one for an accessory left renal artery.

Except for one upward-facing branch, every branch was bridged to the target vessel with at least one balloon-expandable covered stent.

Most commonly (52.6%), a combination of balloon-expandable and self-expandable was used. Only in one case we used just a self-expandable stent for bridging.

In 57.9% of the cases, more than one BSG was used for the upward-facing branch.

The mean branch diameter was 6 mm ± 0.47, and the most common diameter of the BSGs was 6 mm (25, 75.8%), while seven BSGs had a diameter of 5 mm (21.2%) and one a diameter of 7 mm (3.0%). Five of the overall eight balloon-expandable covered stents (62.5%) for the right renal artery had a length of 59 mm. All procedures combined, 12 balloon-expandable covered stents were used for the left renal artery. Six of them (50.0%) had a length of 59 mm, four (33.3%) had a length of 79 mm, and two (16.7%) had a length of 39 mm. Every used self-expandable covered stent was 50 mm long.

Detailed information about the upward-facing branches and the characteristics of the target vessels and BSGs is presented in Table 3.

Technical success was 100%. No stent dislocation or primary stent occlusion was reported.

The mean follow-up was 14.1 ± 12.5 months. A postoperative upward-facing branch-related complication during the hospital stay was reported for one patient (5.9%). The patient had a BEVAR with five branches because there were two similar-sized right renal arteries. One of them was connected through a downward-facing branch and the other through an upward-facing branch. The upward-facing branch was bridged to the right renal artery with a 6 × 39 mm balloon-expandable covered stent and a 5 × 50 mm self-expandable covered stent, while the downward-facing branch was bridged with a 6 × 79 mm balloon-expandable stent. The target vessel had a diameter of approximately 4.5 mm. Final angiography of the procedure showed technical success and no sign of occlusion, dissection, or bleeding. During the postoperative monitoring in our ICU, the patient mentioned pain of the right back and flank and showed a loss of hemoglobin and an increase in lactate. Computed tomography angiography showed bleeding of the right renal arteries close to the kidney. The emergency angiography showed bleeding from small branches of both right renal arteries. Both branches were embolized with Onyx. After that, no further bleeding was detected, and the patient was stable and showed no other complications during the hospital stay.

In an elective follow-up computed tomography angiography approximately six months after the intervention, an occlusion of an upward-facing branch for the left renal artery was detected. This patient was treated with custom-made BEVAR with four branches. The branches for the right and the left renal arteries were planned as upward-facing branches. Both were bridged from a transfemoral access. Preoperative CTA showed a diameter of about 5.0 mm of the renal arteries on both sides. The right renal artery was bridged with two balloon-expandable covered stents (6 × 59 mm and 6 × 29 mm) and one self-expandable covered stent (6 × 50 mm). The left renal artery was bridged with two balloon-expandable and two self-expandable covered stents in total. We started with two 6 × 39 mm balloon expendable covered stents and one self-expandable covered stent (5 × 50 mm). After the deployment of the three stents, a dissection of the target vessel occurred in angiography. This complication necessitated the extension of the BSG to ensure vessel integrity and patency. To cover the dissection, another self-expandable covered stent (5 × 50 mm) was implanted. The final angiography showed a good result. Also, the in-hospital CTA some days after the procedure showed no sign of occlusion, dissection, or other upward-facing branch-related complications. As mentioned above, the first elective CTA after hospital discharge showed an occlusion of the left renal artery upward-facing branch, while the upward-facing branch of the right renal artery was patent, underscoring the technical challenges associated with such anatomies. As far as we know, there was no event of abdominal pain or flank pain and no abnormal lab values.

In one case, there was a total loss of follow-up after discharge from the hospital. Except for the mentioned bleeding and the branch occlusion, no other upward-facing branch-related complications were reported.

We reached a six-month follow-up in 14 patients (82.3%). One year follow-up could be achieved in 41.2% (seven patients) of the study cohort. After one year and until loss of follow-up, there were also no further upward-facing branch-related complications.

## 4. Discussion

The use of upward-facing branches for endovascular treatment of thoracoabdominal aneurysm repair (BEVAR) represents a significant development in the management of complex thoracoabdominal aortic aneurysms. BEVAR requires complex access routes and variable device configurations, including outer branches, inner branches, and fenestrations. However, upward-facing branches offer a streamlined, transfemoral-only approach that could simplify procedures, particularly for patients for whom upper-extremity access is not ideal. They could also help connect cranially oriented target vessels [1].

Eliminating the need for axillary access could reduce operative time and, theoretically, minimize the risk of stroke and lower the rate of access site complications [6]. In our cohort, branched devices with downward-oriented branches were used, necessitating transaxillary access and thus biasing the possible reduced risk of stroke. Ferreira et al. demonstrated the feasibility of this method using custom-made inner branches facing cranially in thoracic arch repairs, achieving technical success and favorable early outcomes without the need for access to the upper extremities [8]. This design simplifies cannulation when approached from below, which is particularly beneficial in patients with tortuous anatomy, where maneuverability from above may be limited.

Avoiding access to the upper extremities may help operators reduce the risks associated with navigating calcified vessels or managing embolic complications that can arise from arch manipulation [9]. Such complications can increase morbidity and the length of hospital stay.

As for every endovascular branched repair, misalignment of the branch can lead to failed cannulation, branch occlusion, or persistent endoleaks [4]. However, this is a problem that is more likely to be encountered during fenestrated aortic repair. The technique for upward-facing branches is the same as for downward branches: by correctly rotating the graft and leaving a distance of 1.5 to 2 cm between the outer ring of the branch and the ostium of the target vessel, cannulation is straightforward. In our cohort, all target vessels could be correctly cannulated, achieving 100% technical success. To avoid canulation failures, branch angulation was assessed with the preoperative planning.

Another consideration is that, in the case of custom-made devices, it is possible to plan two branches for the same target vessel, one downward- and the second upward-oriented, to elevate the chances of connecting the branch. This redundancy may provide added safety in complex anatomies and reduce the need for secondary interventions.

Studies analyzing the performance of upward-facing branches in BEVAR for thoracoabdominal aortic aneurysms are missing; but this technology is used widely for arch branch devices [8]. Comparative studies between branched and fenestrated approaches indicate that branches may have a slightly increased risk of visceral vessel loss over time. Renal arteries, in particular, were more prone to occlusion in branched configurations compared to fenestrated ones [10]. This may relate to hemodynamic factors at the branch–stent interface or technical difficulties during cannulation and stent deployment. Nevertheless, the combination of balloon-expandable covered stents and self-expandable covered stents for the connection of the branches seems to be related to better outcomes for the renal arteries when compared to balloon-expandable stents alone, with patency rates at three years reaching 96% [5].

Continual refinement of branched endograft designs has made upward-facing branches more adaptable to different anatomies. Custom-made devices now incorporate modular configurations, variable angulations, and enhanced radiopaque markers to improve alignment accuracy [11]. Supported by 3D modeling and virtual simulation, procedural planning has advanced to allow “virtual rehearsals”, significantly reducing intraoperative uncertainty [12].

As experience and confidence in this technique increases, the use of upward-facing branches is likely to grow. To the best of the authors’ knowledge, this is the first early-feasibility study about using upward-facing branches, marking a shift towards fully endovascular solutions for traditionally open surgical pathologies.

A limitation of this study is the retrospective nature and the small number of patients. There is also a variability of the duration of follow-up.

## 5. Conclusions

In this small cohort, upward-facing branches in BEVAR offered a valuable alternative to traditional fenestrated or directional branches by enabling exclusive transfemoral access and minimizing procedural risks. The results of this study suggest that upward-facing branches may be a safe and effective option in the treatment of complex thoracoabdominal aortic aneurysms, offering technical advantages and potential clinical benefits. As more centers gain expertise and new data emerges, upward-facing branches will likely become a cornerstone of complex aortic aneurysm management in selected patients. But this must be proven in studies with bigger cohorts and long-term data.

## Figures and Tables

**Table 1:** Demographics and comorbidities of the patients.

	Total
Number of patients (%)	17 (100)
Age, mean	70.29
(SD, min–max)	(±6.06, 62–82)
Sex, male n (%)	10 (59)
Comorbidities, risk factors, and prior treatment, n (%)	
Hypertension	15 (88)
Smoking	
History	3 (18)
Active	1 (6)
DM Type II	2 (12)
Coronary artery disease	8 (47)
Peripheral artery disease	1 (18)
Chronic kidney disease	3 (18)
COPD	5 (29)
Dyslipidemia	8 (47)
Previous aortic repair	7 (41)
TEVAR	6 (35)
FET	2 (12)

TEVAR: Thoracic endovascular aortic repair; FET: Frozen elephant trunk.

**Table 2:** Anatomic information and number of target vessels.

		Aortic Pathology					Total
		TAAA				Other (PAU)	
		II	III	IV	V		
Total, N (%)		6 (35.3)	3 (17.8)	6 (35.3)	1 (5.8)	1 (5.8)	17 (100)
Max. aneurysm diameter, median (SD, min–max)		60.5 (±2.05, 59–65)	65 (±6.13, 52–65)	60.5 (±6.69, 43–62)	60	38	60 (±7.24, 38–65)
Number of target vessels							
	4-branch EVAR	6 (100)	1 (33)	5 (83)	0 (0)	1 (100)	13 (76.5)
	5-branch EVAR	0 (0)	2 (67)	1 (17)	1 (100)	0 (0)	4 (23.5)

TAAA: thoracoabdominal aortic aneurysm; PAU: penetrating aortic ulcer.

**Table 3:** Upward-facing branches and characteristics of TV and BSG.

**Number and location of upward-facing branches**					
1 branch		15 (88.2)			
RRA		4 (23.5)			
LRA		10 (58.8)			
Accessory LRA		1 (5.9)			
2 branches		2 (11.8)			
		**Target Vessel**			Total
		RRA	LRA	Accessory LRA	
Total, N (%)		6 (31.6)	12 (63.2)	1 (5.2)	19 (100)
Target vessel diameter in mm, mean (SD, min–max)		4.97 (±0.32, 4.5–5.5)	5 (±0.43, 4.2–6)	4	4.94 (±0.45, 4–6)
Stenosis >50%, n (%)		0 (0)	2 (16.7)	0 (0)	2 (10.5)
Dissection, n (%)		1 (16.7)	1 (8.3)	0 (0)	2 (10.5)
Branch diameter in mm, median (SD, min–max)		6	6 (±0.49, 5–7)	6	6 (±0.47, 5–7)
Number of bridging stents used, n (%)					
	1 stent	2 (33.3)	6 (50)	0 (0)	8 (42.1)
	2 stents	3 (50)	5 (41.7)	1 (100)	9 (47.4)
	3 stents	1 (16.7)	0 (0)	0 (0)	1 (5.25)
	4 stents	0 (0)	1 (8.3)	0 (0)	1 (5.25)
Length of stent used (VBX), n (%)		(N = 8)	(N = 12)	(N = 1)	(N = 21)
	29 mm	1 (12.5)	0 (0)	0 (0)	1 (4.8)
	39 mm	1 (12.5)	2 (16.7)	0 (0)	3 (14.3)
	59 mm	5 (62.5)	6 (50)	1 (100)	12 (57.1)
	79 mm	1 (12.5)	4 (33.3)	0 (0)	5 (23.8)
Length of stent used (Viabahn self-expandable), n (%)		(N = 3)	(N = 8)	(N = 1)	(N = 12)
	50 mm	3 (100)	8 (100)	1 (100)	12 (100)
Total length of stents used, in mm, median (SD, min–max)		99 (±32.7, 59–138)	94 (±31.8, 50–178)	109	109 (±31.5, 50–178)

RRA: right renal artery; LRA: left renal artery; VBX: Viabahn VBX Ballon expandable ballon endoprothesis by W.L. Gore & Associates (Flagstaff, AZ, USA); Viabahn by W.L. Gore & Associates (Flagstaff, AZ, USA).

## Data Availability

The presented data is available on request from the corresponding author.
